# Influence of Onset to Imaging Time on Radiological Thrombus Characteristics in Acute Ischemic Stroke

**DOI:** 10.3389/fneur.2021.693427

**Published:** 2021-06-18

**Authors:** Manon L. Tolhuisen, Manon Kappelhof, Bruna G. Dutra, Ivo G. H. Jansen, Valeria Guglielmi, Diederik W. J. Dippel, Wim H. van Zwam, Robert J. van Oostenbrugge, Aad van der Lugt, Yvo B. W. E. M. Roos, Charles B. L. M. Majoie, Matthan W. A. Caan, Henk A. Marquering, the MR CLEAN Registry Investigators

**Affiliations:** ^1^Department of Biomedical Engineering and Physics, Amsterdam University Medical Centers, University of Amsterdam, Amsterdam, Netherlands; ^2^Department of Radiology and Nuclear Medicine, Amsterdam University Medical Centers, University of Amsterdam, Amsterdam, Netherlands; ^3^Nico.Lab B.V., Amsterdam, Netherlands; ^4^Department of Neurology, Amsterdam University Medical Centers, University of Amsterdam, Amsterdam, Netherlands; ^5^Department of Neurology, Erasmus Medical Center University Medical Center, Rotterdam, Netherlands; ^6^Department of Radiology and Nuclear Medicine, Maastricht University Medical Center, Maastricht, Netherlands; ^7^Department of Neurology, Maastricht University Medical Center, Maastricht, Netherlands; ^8^Cardiovascular Research Institute Maastricht, University of Maastricht, Maastricht, Netherlands; ^9^Department of Radiology and Nuclear Medicine, Erasmus Medical Center University Medical Center, Rotterdam, Netherlands

**Keywords:** ischemic stroke, endovascular treatment, radiological thrombus characteristics, acute ischemic stroke, computed tomography, thrombus perviousness, thrombus length, thrombus density

## Abstract

**Introduction:** Radiological thrombus characteristics are associated with patient outcomes and treatment success after acute ischemic stroke. These characteristics could be expected to undergo time-dependent changes due to factors influencing thrombus architecture like blood stasis, clot contraction, and natural thrombolysis. We investigated whether stroke onset-to-imaging time was associated with thrombus length, perviousness, and density in the MR CLEAN Registry population.

**Methods:** We included 245 patients with M1-segment occlusions and thin-slice baseline CT imaging from the MR CLEAN Registry, a nation-wide multicenter registry of patients who underwent endovascular treatment for acute ischemic stroke within 6.5 h of onset in the Netherlands. We used multivariable linear regression to investigate the effect of stroke onset-to-imaging time (per 5 min) on thrombus length (in mm), perviousness and density (both in Hounsfield Units). In the first model, we adjusted for age, sex, intravenous thrombolysis, antiplatelet use, and history of atrial fibrillation. In a second model, we additionally adjusted for observed vs. non-observed stroke onset, CT-angiography collateral score, direct presentation at a thrombectomy-capable center vs. transfer, and stroke etiology. We performed exploratory subgroup analyses for intravenous thrombolysis administration, observed vs. non-observed stroke onset, direct presentation vs. transfer, and stroke etiology.

**Results:** Median stroke onset-to-imaging time was 83 (interquartile range 53–141) min. Onset to imaging time was not associated with thrombus length nor perviousness (β 0.002; 95% CI −0.004 to 0.007 and β −0.002; 95% CI −0.015 to 0.011 per 5 min, respectively) and was weakly associated with thrombus density in the fully adjusted model (adjusted β 0.100; 95% CI 0.005–0.196 HU per 5 min). The subgroup analyses showed no heterogeneity of these findings in any of the subgroups, except for a significantly positive relation between onset-to-imaging time and thrombus density in patients transferred from a primary stroke center (adjusted β 0.18; 95% CI 0.022–0.35).

**Conclusion:** In our population of acute ischemic stroke patients, we found no clear association between onset-to-imaging time and radiological thrombus characteristics. This suggests that elapsed time from stroke onset plays a limited role in the interpretation of radiological thrombus characteristics and their effect on treatment results, at least in the early time window.

## Introduction

Radiological thrombus characteristics are among the few biomarkers that are associated with acute ischemic stroke (AIS) treatment success. Thrombus perviousness, reflecting the extent to which intravenous contrast permeates into a thrombus, was shown to be strongly associated with higher recanalization rates and treatment success of intravenous alteplase (IVT) (1, 2). Thrombus length was reported to negatively affect success rates of both IVT and endovascular treatment (EVT) (3, 4), although no effect on EVT outcomes was found in some other studies (5, 6). Higher thrombus density is related to higher recanalization rates after IVT and EVT (7, 8).

Thrombus characteristics may vary over time. For example, stasis in low pressure systems can cause thrombus growth over time by the accumulation of red blood cells in low-density fibrin networks (9). In contrast, time may allow for natural thrombolysis or IVT to reduce the size of the clot (10–13). In addition, if a patient has good collaterals, decreased blood stasis was reported to limit thrombus growth distal to the clot and improve thrombus exposure to alteplase (14, 15). Clot contraction may also reduce thrombus length, increase thrombus density, and decrease perviousness (16, 17).

Dynamic behavior of thrombi may influence the success of stroke treatment. For example, patients with a prolonged time to AIS treatment and favorable thrombus dynamics may show alteplase-induced or even spontaneous recanalization. This effect has been observed in patients transferred from primary hospitals to comprehensive stroke centers for EVT (18). Alternatively, if the thrombus grows before treatment, the chance of recanalization with IVT reduces, and endovascular procedure time increases (3, 4). Moreover, if radiological thrombus characteristics change over time, elapsed time between the moment of measurement and the start of stroke treatment may affect the association between these values and stroke treatment outcomes.

Despite these possibly relevant effects, the effects of time on thrombus characteristics have been understudied. We therefore aimed to assess the relation between stroke onset to imaging time and thrombus length, perviousness, and density using data from a large national registry.

## Methods

### Study Population

This study includes patients from the Multicenter Randomized Clinical trial of Endovascular Treatment for Acute ischemic stroke in the Netherlands (MR CLEAN) Registry (part I) (19) between March 2014 and June 2016. The MR CLEAN Registry is a nation-wide, prospective, observational, multicenter study at 16 comprehensive stroke centers in the Netherlands, including all patients who underwent EVT for AIS since the completion of the MR CLEAN trial (20). IVT was administered before EVT if patients were eligible. The central medical ethics committee of the Erasmus Medical Center Rotterdam, the Netherlands, granted permission (MEC-2014–235) to perform the study as a registry. Source data of this study are available in anonymized form upon reasonable request to the corresponding author.

Inclusion criteria for the current study were: M1 occlusion; age ≥18 years; groin puncture within 6.5 h after stroke onset; and treatment in an MR CLEAN trial center. Only patients with thin-slice (≤2.5 mm) CT-angiography (CTA) and non-contrast CT (NCCT) images that were acquired on the same scanner no longer than 30 min apart were included. We used the images acquired at the first point in time. For patients who were transferred from a primary stroke center we used the primary center's radiological images if they were available and of sufficient quality. Otherwise, we used the images acquired at the comprehensive stroke center. Patients were excluded if images contained excessive noise, artifacts, poor contrast opacification on CTA, or uncorrectable registration errors. Patients with calcified thrombi were excluded as well, since the high attenuation of these thrombi can cause streak and partial volume artifacts.

### Image Analysis

Measurements of radiological thrombus characteristics were performed in ITK-SNAP (www.itksnap.org) (19) by two neuroradiologists (B.G.D. and H.A.) (4). The NCCT and CTA images for each patient were co-registered with rigid registration, using Elastix® (21), such that thrombus measurements could be performed in both modalities simultaneously. If alignment of the CTA and NCCT was suboptimal, we performed manual registration.

Thrombus length was measured manually using the ITK-SNAP ruler function (22). If contrast pick-up distal to the thrombus was not seen on CTA, the hyperdense artery sign on NCCT was used as a reference point for the distal thrombus end. If the thrombus extended into two arterial branches, the longest thrombus length was included as measurement.

Thrombus perviousness and density were computed from three region of interests (ROIs). On the co-registered NCCT and CTA images, three spherical ROIs with a 1 mm radius were placed in the proximal, middle, and distal parts of the thrombus. Thrombus density was defined as the mean density of these ROIs on NCCT, in Hounsfield Units (HU). Thrombus perviousness was computed by subtracting the mean density of the ROIs on NCCT from the mean density of the ROIs on CTA, resulting in the average thrombus attenuation increase in HU (thrombus perviousness = ρ_CTA_ – ρ_NCCT_).

Collateral score (23), occlusion location, Alberta Stroke Program Early CT Score were assessed on baseline CTA and NCCT by the MR CLEAN Registry core laboratory (19).

### Statistical Analysis

The dependent variables were thrombus length (mm), perviousness and density (HU). The independent variable of interest was time from symptom onset or last seen well to imaging per 5 min. Imaging time was defined as the acquisition time of the NCCT images. Baseline characteristics were summarized appropriate to the type of data. Comparisons were made by one-way ANOVA, Kruskal-Wallis, Mann-Whitney-*U*, or Fisher's exact-test appropriate to the type of data. Visual representations of the data were made with scatter and bar plots.

Univariable and multivariable linear regression were used to assess the association between onset to imaging time and thrombus length, perviousness, and density, resulting in beta coefficients (β) with 95% confidence intervals (95% CI). The multivariable models were adjusted for the following baseline pre-specified variables: age, sex, history of atrial fibrillation, IVT administration, and antiplatelets. Model 2 was additionally adjusted for: observed stroke vs. non-observed stroke, CTA collateral score, transfer or direct presentation at a comprehensive stroke center, and stroke etiology according to the modified Trial of ORG 10172 in Acute Stroke Treatment (TOAST) criteria (cardio-embolic vs. large artery atherosclerosis vs. unknown). The TOAST criteria were scored for a previous study on our data set (15). Because thrombus length and perviousness showed a right-skewed distribution, they were log-transformed for the regression analyses (Supplementary Figure 1).

Exploratory sensitivity analyses were performed by comparing the results of univariable models for different subgroups: (a) patients with observed stroke onset vs. patients without observed stroke onset (using last-seen-well time as onset time), (b) patients with vs. without IVT administration prior to EVT, (c) patients with collateral score 0–1 vs. patients with CS 2–3, (d) transfer patients vs. direct presentation to a comprehensive stroke center, (e) patients with different stroke etiologies: cardioembolic stroke, large-artery atherosclerotic stroke and stroke with an undetermined origin.

Missing data in the main and secondary variables of interest were imputed using multiple imputation for regression analyses only, based on relevant covariates and outcomes. A two-sided *p*-value of 0.05 was considered significant. Statistical analyses were performed with Stata/SE 14.2 (StataCorp, TX).

## Results

**Figure 1:**
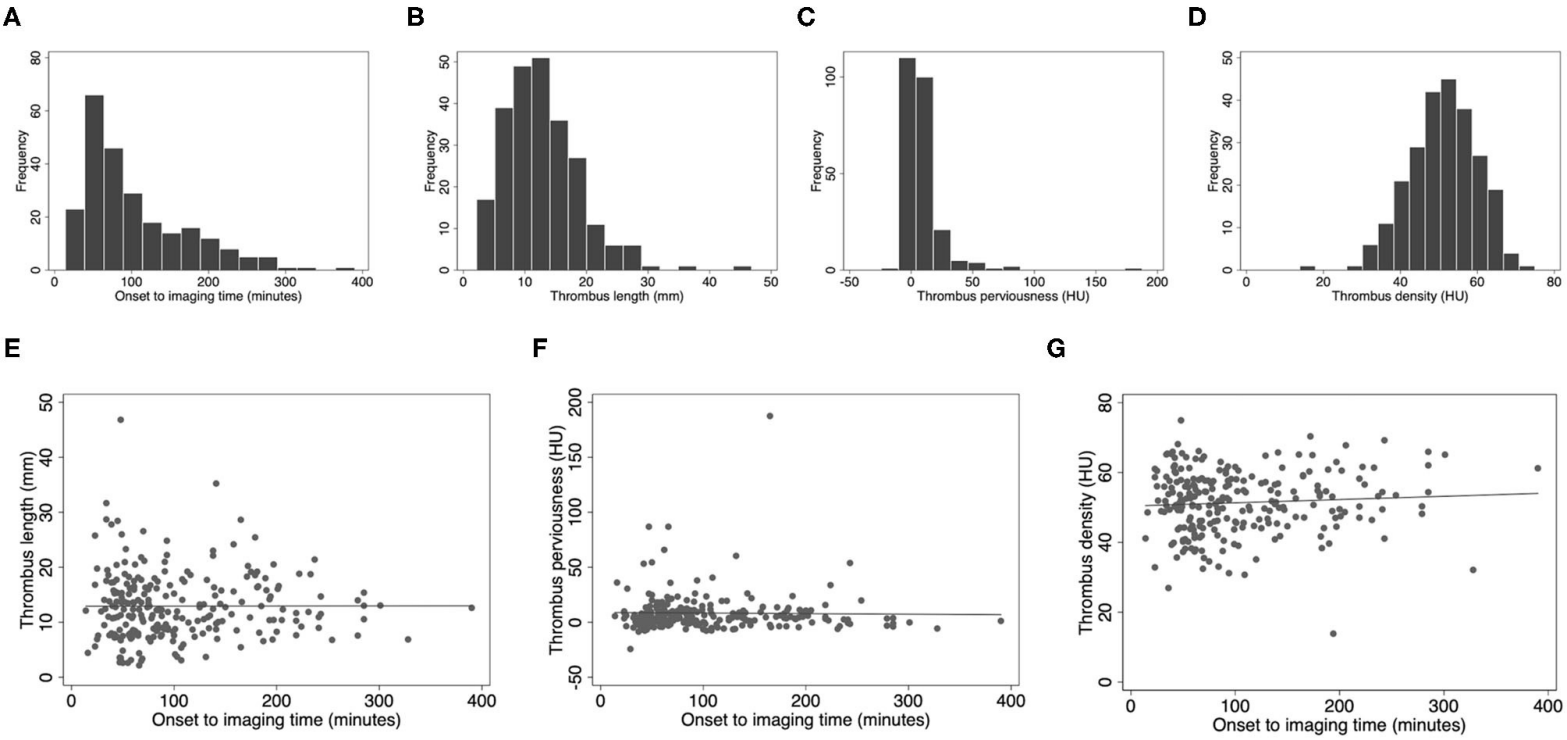
Scatter and box plots of image characteristics. **(A)** Time from symptom onset to imaging distribution, **(B)** thrombus length histogram, **(C)** thrombus perviousness histogram, **(D)** thrombus density histogram, **(E)** onset to imaging time vs. thrombus length, **(F)** onset to imaging time vs. perviousness, **(G)** onset to imaging time vs. non-contrast CT density. HU, Hounsfield Units; mm, millimeter.

The total MR CLEAN Registry part I population consisted of 1,627 patients, of whom 825 had an M1 occlusion. We included 245 patients in the current study (Supplementary Figure 2 and Table 1). Of these, 90 patients were transferred from a primary to a comprehensive center for EVT. We measured radiological thrombus characteristics on images acquired in the primary center for 44 of these patients. Baseline characteristics of our study population were similar to the overall MR CLEAN Registry population with an M1 occlusion except for a lower frequency of patients transferred from a primary stroke center [90/245 (36%) vs. 441/825 (53%), *p* < 0.01]. Median time from stroke onset to imaging was 83 (IQR 53–141) min. Median thrombus length was 12 (IQR 9–16) mm, median perviousness was 5 (IQR 0.1–11) HU, and median density was 52 (IQR 46–58) HU (Figures 1A–D). Figures 1E–G show the values of onset to imaging time in relation to thrombus length, thrombus perviousness and thrombus density for all patients.

**Table 1:** Baseline characteristics of patients included in the current study, compared to all MR CLEAN Registry patients with an M1 occlusion.

	**Current study** ** (*n* = 245)**	**MR CLEAN Registry patients with M1 occlusion (*n* = 825)**	***P***
**Baseline clinical variables (data known in** ***n*****=)**			
Age, median (IQR)	69 (61–80)	72 (61–80)	0.57
Sex (men), *n* (%)	127 (52)	423 (51)	0.89
NIHSS baseline, median (IQR)	15 (11–20) (243)	16 (11–19) (811)	0.85
SBP, mmHg, median (IQR)	148 (130–162) (238)	150 (131–165) (803)	0.29
Medical history, *n* (%)			
Diabetes mellitus	45 (19) (242)	151 (18) (820)	0.93
Previous stroke	37 (15) (242)	152 (19) (820)	0.29
Atrial fibrillation	48 (20) (240)	195 (24) (812)	0.22
Pre-stroke mRS, *n* (%)			0.45
0–2	204 (85) (240)	707 (86) (814)	
≥3	36 (15) (240)	107 (14) (814)	
**Workflow**			
Observed onset time, *n* (%)	187 (76)	618 (75)	0.67
Intravenous alteplase, *n* (%)	188 (77)	637 (78)	0.86
Transferred from primary stroke center^*^, *n* (%)	90 (36)	441 (53)	<0.01
Time from onset to presentation at first hospital, minutes, median (IQR)	55 (40–92) (200)	55 (39–93) (640)	0.87
Time from onset to imaging^$^, minutes, median (IQR)	83 (53–141)	69 (51–106) (733)	0.62
**Imaging variables**			
ASPECTS subgroups, *n* (%)			0.47
0–4	9 (4)	46 (6)	
5–7	56 (23)	186 (23)	
8–10	180 (73)	571 (71)	
Collateral score, *n* (%) (known in)			0.95
0	16 (7) (240)	48 (6)	
1	72 (30) (240)	252 (31)	
2	97 (40) (240)	323 (40)	
3	55 (23) (240)	179 (22)	
Extracranial carotid tandem lesion^#^	28 (11) (192)	136 (16) (689)	0.14
Thrombus length, mm, median (IQR)	12 (9–16)	NA	NA
NCCT thrombus density, HU, median (IQR)	52 (46–58)	NA	NA
Thrombus perviousness, attenuation increase, HU, median (IQR)	5 (0.1–11)	NA3	NA

*ASPECTS, Alberta Stroke Program; Early CT Score; CTA, CT-angiography; IQR, interquartile range; HU, Hounsfield Units; mRS, modified Rankin Scale; NA, not applicable; NCCT, non-contrast CT; NIHSS, National Institutes of Health Stroke Scale; SBP, systolic blood pressure. If no (known in) number is shown, data were available for all included patients*.

* *Images from the primary stroke center were used in 44/90 transfer patients (49%)*.

$ *In current study sample: time of imaging used for measurements. In all Registry M1 occlusion patients: time of first acquired imaging*.

# *Tandem lesion was defined as an atherosclerotic occlusion, high-grade stenosis, or dissection ipsilateral to the intracranial occlusion, as assessed on baseline CT angiography*.

The regression coefficients of the association of onset-to-imaging time and thrombus length, perviousness, or density are presented in Table 2. None of these associations were statistically significant, except for a positive association for thrombus density in the adjusted Model 2 (β 0.10; 95% CI 0.005–0.20 HU/5 min, Table 2). The sensitivity analyses showed no statistically significant associations for thrombus length, perviousness, or density in any of the subgroups (Supplementary Tables 1–4 and Supplementary Figures 3–7), except for a significantly positive relation between onset-to-imaging time and thrombus density in patients transferred for EVT from a primary stroke center (*n* = 90) in the adjusted Model 2 only (β 0.18; 95%CI 0.022–0.35 HU/5 min, Supplementary Table 4). Patients who were transferred from a primary center had longer median onset to imaging times (median 137 min, IQR 65–181) than those presented directly to a comprehensive center (median 69 min, IQR 48–103, *p* < 0.01). In addition, among IVT-treated transferred patients (*n* = 77), median onset-to-imaging times were shorter among patients whose thrombus characteristics were measured on images acquired in the primary stroke center (*n* = 36; 67 min, IQR 56–100), as compared to the comprehensive stroke center (*n* = 41, 175 min, IQR 138–197; *p* < 0.01). Nonetheless, the longer time for IVT to work did not affect the association between onset-to-imaging time and thrombus characteristics (Supplementary Table 4).

**Table 2:** Beta coefficients of the effect of time from stroke onset to CT imaging (per 5 min) on thrombus characteristics.

**Outcome variable**	**Model 0**		**Model 1**		**Model 2**	
	**Unadjusted**		Adjusted for pre-specified variables^*^		Adjusted for pre-specified variables^*^+ **variables of interest^#^**	
	**β**	**95% CI**	**β**	**95% CI**	**β**	**(95% CI)**
Thrombus length	0.002	−0.002 to 0.007	0.003	−0.002 to 0.008	0.002	−0.004 to 0.007
Perviousness	−0.005	−0.012 to 0.011	−0.001	−0.012 to 0.012	−0.002	−0.015 to 0.011
Thrombus density	0.046	−0.036 to 0.129	0.047	−0.035 to 0.120	**0.100**	**0.005 to 0.196**

*CI, confidence interval; ICA-T, internal carotid artery terminus*.

* *Pre-specified variables: age, sex, and history of atrial fibrillation*.

# *Variables of interest: observed stroke onset, intravenous alteplase, CTA collateral score, direct presentation at thrombectomy-capable center or transfer, stroke etiology (cardio-embolic vs. large artery atherosclerosis vs. unknown). Values printed in bold are statistically significant (*p* < 0.05)*.

## Discussion

Our study showed no association between stroke-onset to imaging time and thrombus length, density and perviousness, suggesting that within the critical time window of treatment no observable changes occur. Thrombus density may slightly increase over time, which was visible in our data in patients transferred from a primary stroke center. Transferred patients had a longer median onset to imaging time, possibly allowing for a higher density difference to develop. This density increase could be caused by the contraction of the thrombus resulting in the compression of erythrocytes in a densely packed structure, though may also have been a chance finding (17). Overall, however, the effects of thrombus contraction (16, 17), thrombus growth (9), and endogenous or alteplase-induced thrombolysis (10–13) seem to balance each other out in the time window we observed.

Only a small number of studies have been reported that focus on the influence of time on thrombus image characteristics. Qazi et al. (24) included onset to imaging time for the analysis of thrombus characteristics in patients with AIS. They have studied the relation between collateral status and thrombus length. Similar to our study, onset-to-imaging time did not influence thrombus length. Also, Pikija et al. (25) have studied the relation of time with thrombus density. In contrast to our results, their results showed a drop in thrombus density within a 5-h time window for onset to imaging time. Finally, Haridy et al. (26) reported no association between the presence of a hyperdense artery sign (HAS) or relative thrombus density and onset to imaging time within a 24 h time window. Unfortunately, they did not specifically study the relation of time with thrombus density or perviousness. Therefore, we cannot directly compare our results with their study.

Since the assessment of the radiological thrombus characteristics addressed in this study is not part of current treatment decision making in clinical practice and is not included in the national or international stroke guidelines (27), our results do not give rise to changes in the standard clinical care for AIS. For research on radiological thrombus characteristics in relation to stroke treatment outcomes, our results indicate that the elapsed time from symptom onset is of limited influence on the values of these characteristics, and as such would not have to be taken into account in the time window that we investigated.

Our study has limitations. First, a selective group of patients was included. Our study population contained patients who underwent EVT and therefore included severe cases of stroke only. All patients were treated within a short time window, since the onset to hospital time is relative low due to the small surface area and high hospital density of the Netherlands (19, 28). In addition, it is expected that the treatment window for EVT will be extended in the future and onset to imaging time will be prolonged. Increased variation in time from stroke onset may make changes in radiological thrombus characteristics more pronounced (29). In the overall Registry population, the proportion of transfer patients was higher than in our study sample. This may have contributed to our shorter median onset to imaging time: thin-slice CT scans are less often available for transferred patients, which was one of our inclusion criteria. Second, the dynamic behavior of thrombus size could not be assessed in a controlled environment; we combined data of a heterogeneous group of patients. To reduce the variability, we only selected patients with an occlusion of the M1, though this resulted in a relatively small sample size. Third, thrombus measurements were performed on single-phase CTA. As such, results are dependent on the phase of the CTA. In case of stasis of blood flow and early CTA san timing, the contrast may not reach the exact proximal location of the thrombus, and contrast may not have reached the distal part of the thrombus. This may have resulted in an overestimation of thrombus length and lower perviousness values. Future implementation multiphase CTA may resolve that issue (30). Fourth, we tried to assess the dynamic behavior of thrombi on imaging made at a single point in time. Ideally, thrombus measurements would be performed at two moments in time in the same patient, to address individual rates of thrombus growth or shrinkage. By comparing thrombus characteristics in a large group of patients with varying onset-to-imaging times, we expected other factors influencing thrombus length to be approximately evenly distributed. Fifth, thrombi may be older than the duration of stroke symptoms, and hence be more organized than what one would expect based on the time from stroke onset to imaging. Cardiac thrombi for example may form and age in the heart, break loose, and embolize to cause a stroke (31, 32). However, our results did not vary between stroke etiology subgroups. Sixth, apparent trends in the subgroup analyses may not have translated to statistically significant regression results due to the small number of patients in the subgroups. However, our effect estimates were close to zero and any trends found in the data visualization may have occurred due to chance. Finally, because we only included patients with an M1-occlusion to improve data homogeneity, we could not assess differences in thrombus location and length. Thrombi may contract over time in all directions, instead of only in length, thereby decreasing in diameter and embolizing to a more distal location. Further research with more observations in distal occlusion locations could focus on the association between onset-to-imaging time and the distance from the carotid terminus to the proximal thrombus border.

## Conclusion

Our results did not show a clear association between onset to imaging time and radiological thrombus characteristics for AIS patients within the observed time window. Only thrombus density slightly increased with longer onset to imaging time intervals due to interhospital transfer. There was no association between time and thrombus perviousness or length. This suggests that elapsed time from stroke onset plays a limited role in the interpretation of radiological thrombus characteristics and their effect on treatment results, at least in the relatively short time window observed in this study.

## Data Availability Statement

The datasets presented in this article are not readily available because of patient consent restrictions for reuse of data, but analysis code and results are available upon reasonable request to the corresponding author. Requests to access the datasets should be directed to MK, m.kappelhof@amsterdamumc.nl.

## Ethics Statement

The studies involving human participants were reviewed and approved by Erasmus Medical Center Rotterdam, the Netherlands (MEC-2014–235). The patients/participants provided their written informed consent to participate in this study.

## Author Contributions

MT, MK, IJ, and BD collected the data. MT, MK, VG, HM, and MC conceived the study idea and conceptualized the analysis. MK, MT, HM, and MC wrote the manuscript and performed the statistical analyses. All authors discussed the ideas and results and critically revised the manuscript.

## Conflict of Interest

Erasmus MC received funds from Stryker by AL. Amsterdam UMC received funds from Stryker for consultations by CM and YR. MUMC received funds from Stryker and Codman for consultations by WZ. CM reports grants from the TWIN Foundation, the CVON/Dutch Heart Foundation, the European Commission. HM is cofounder and shareholder of Nico.lab. CM, MC, and YR own stock in Nico.lab. The remaining authors declare that the research was conducted in the absence of any commercial or financial relationships that could be construed as a potential conflict of interest.

## References

[1] Santos EMM Dankbaar JW Treurniet KM Horsch AD Roos YB Kappelle LJ. Permeable thrombi are associated with higher intravenous recombinant tissue-type plasminogen activator treatment success in patients with acute ischemic stroke. Stroke. (2016) 47:2058–65. 10.1161/STROKEAHA.116.013306 27338928

[2] Santos EMM Marquering HA den Blanken MD Berkhemer OA Boers AMM Yoo AJ. Thrombus permeability is associated with improved functional outcome and recanalization in patients with ischemic stroke. Stroke. (2016) 47:732–41. 10.1161/STROKEAHA.115.011187 26846859

[3] Riedel CH Zimmermann P Jensen-Kondering U Stingele R Deuschl G Jansen O. The importance of size: successful recanalization by intravenous thrombolysis in acute anterior stroke depends on thrombus length. Stroke. (2011) 42:1775–7. 10.1161/STROKEAHA.110.609693 21474810

[4] Dutra BG Tolhuisen ML Alves CBRH Treurniet KM Kappelhof M Yoo AJ. Thrombus imaging characteristics and outcomes in acute ischemic stroke patients undergoing endovascular treatment. Stroke. (2019) 50:2057–64. 10.1161/STROKEAHA.118.024247 31216961

[5] Weisstanner C Gratz PP Schroth G Verma RK Köchl A Jung S. Thrombus imaging in acute stroke: correlation of thrombus length on susceptibility-weighted imaging with endovascular reperfusion success. Eur Radiol. (2014) 24:1735–41. 10.1007/s00330-014-3200-3 24832928 PMC4082654

[6] Seker F Pfaff J Wolf M Schönenberger S Nagel S Herweh C. Impact of thrombus length on recanalization and clinical outcome following mechanical thrombectomy in acute ischemic stroke. J Neurointerv Surg. (2017) 9:937–9. 10.1136/neurintsurg-2016-012591 27634955

[7] Moftakhar P English JD Cooke DL Kim WT Stout C Smith WS. Density of thrombus on admission CT predicts revascularization efficacy in large vessel occlusion acute ischemic stroke. Stroke. (2013) 44:243–6. 10.1161/STROKEAHA.112.674127 23111438

[8] Mokin M Morr S Natarajan SK Lin N Sneyder KV Hopkins LN. Thrombus density predicts successful recanalization with Solitaire stent retriever thrombectomy in acute ischemic stroke. J Neurointerv Surg. (2015) 7:104–7. 10.1136/neurintsurg-2013-011017 24510378

[9] De Meyer SF Andersson T Baxter B Bendszus M Brouwer P Brinjikji W. Analyses of thrombi in acute ischemic stroke: a consensus statement on current knowledge and future directions. Int J Stroke. (2017) 12:606–14. 10.1177/1747493017709671 28534706

[10] Docagne F Parcq J Lijnen R Ali C Vivien D. Understanding the functions of endogenous and exogenous tissue-type plasminogen activator during stroke. Stroke. (2015) 46:314–20. 10.1161/STROKEAHA.114.006698 25395410

[11] Zhu J Wan Y Xu H Wu Y Hu B Jin H. The role of endogenous tissue-type plasminogen activator in neuronal survival after ischemic stroke: friend or foe? Cell Mol Life Sci. (2019) 76:1489–506. 10.1007/s00018-019-03005-8 30656378 PMC11105644

[12] Kovacs IB Yamamoto J. Spontaneous thrombolysis: a forgotten determinant of life or death. Clin Appl Thromb. (2006) 12:358–63. 10.1177/1076029606291410 16959691

[13] Minnerup J Kleinschnitz C. Visualization of clot composition in ischemic stroke: do we get what we see? Stroke. (2011) 42:1193–4. 10.1161/STROKEAHA.110.612150 21393600

[14] Alves HC Treurniet KM Dutra BG Jansen IGH Boers AMM Santos EMM. Associations between collateral status and thrombus characteristics and their impact in anterior circulation stroke. Stroke. (2018) 49:391–6. 10.1161/STROKEAHA.117.019509 29321337

[15] Ding X Yan S Zhang R Lou M Zhou Y Zhang S. Slow collateral flow is associated with thrombus extension in patients with acute large-artery occlusion. Am J Neuroradiol. (2018) 39:1088–92. 10.3174/ajnr.A5614 29622554 PMC7410608

[16] Tutwiler V Peshkova AD Andrianova IA Khasanova DR Weisel JW Litvinov RI. Contraction of blood clots is impaired in acute ischemic stroke. Arterioscler Thromb Vasc Biol. (2017) 37:271–9. 10.1161/ATVBAHA.116.308622 27908894 PMC5269459

[17] Cines DB Lebedeva T Nagaswami C Hayes V Massefski W Litvinov RI. Clot contraction: compression of erythrocytes into tightly packed polyhedra and redistribution of platelets and fibrin. Blood. (2014) 123:1596–603. 10.1182/blood-2013-08-523860 24335500 PMC3945867

[18] Kassem-Moussa H Graffagnino C. Nonocclusion and spontaneous recanalization rates in acute ischemic stroke. Arch Neurol. (2002) 59:1870. 10.1001/archneur.59.12.1870 12470173

[19] Jansen IGH Mulder JHLM Goldhoorn RJB. Endovascular treatment for acute ischaemic stroke in routine clinical practice: prospective, observational cohort study (MR CLEAN Registry). BMJ. (2018) 360:k949. 10.1136/bmj.k949 29523557 PMC5844245

[20] Berkhemer OA Fransen PSS Beumer D van den Berg LA Lingsma HF Yoo AJ. A randomized trial of intraarterial treatment for acute ischemic stroke. N Engl J Med. (2014) 372:11–20. 10.1056/NEJMoa1411587 25517348

[21] Klein S Staring M Murphy K Viergever MA Pluim JPW. Elastix: a toolbox for intensity-based medical image registration. IEEE Trans Med Imaging. (2010) 29:196–205. 10.1109/TMI.2009.2035616 19923044

[22] Yushkevich PA Piven J Hazlett HC Smith RG Ho S Gee JC. User-guided 3D active contour segmentation of anatomical structures: significantly improved efficiency and reliability. Neuroimage. (2006) 31:1116–28. 10.1016/j.neuroimage.2006.01.015 16545965

[23] Tan IYL Demchuk AM Hopyan J Zhang L Gladstone D Wong K. CT angiography clot burden score and collateral score: correlation with clinical and radiologic outcomes in acute middle cerebral artery infarct. AJNR. (2009) 30:525–31. 10.3174/ajnr.A1408 19147716 PMC7051470

[24] Qazi EM Sohn SI Mishra S Almekhlafi MA Eesa M d'Esterre CD. Thrombus characteristics are related to collaterals and angioarchitecture in acute stroke. Can J Neurol Sci. (2015) 42:381–8. 10.1017/cjn.2015.291 26365832

[25] Pikija S Magdic J Trkulja V Underkreuter P Mutzenbach JS Novak HF. Intracranial thrombus morphology and composition undergoes time-dependent changes in acute ischemic stroke: a CT densitometry study. Int J Mol Sci. (2016) 17:1–12. 10.3390/ijms17111959 27886084 PMC5133953

[26] Haridy J Churilov L Mitchell P Dowling R Yan B. Is there association between hyperdense middle cerebral artery sign on CT scan and time from stroke onset within the first 24-hours? BMC Neurol. (2015) 15:1–6. 10.1186/s12883-015-0358-5 26133766 PMC4489032

[27] Powers WJ Rabinstein AA Ackerson T Adeoye OM Bambakidis NC Becker K. Guidelines for the early management of patients with acute ischemic stroke: 2019 update to the 2018 guidelines for the early management of acute ischemic stroke a guideline for healthcare professionals from the American Heart Association/American Stroke A. Stroke. (2019) 50:e344–418. 10.1161/STR.0000000000000211 31662037

[28] Goyal M Menon BK van Zwam WH Dippel DWJ Mitchell PJ Demchuk AM. Endovascular thrombectomy after large-vessel ischaemic stroke: a meta-analysis of individual patient data from five randomised trials. Lancet. (2016) 387:1723–31. 10.1016/S0140-6736(16)00163-X 26898852

[29] Kirchhof K Welzel T Mecke C Zoubaa S Sartor K. Differentiation of white, mixed, and red thrombi: value of CT in estimation of the prognosis of thrombolysis phantom study. Radiology. (2003) 228:126–30. 10.1148/radiol.2273020530 12728185

[30] Santos EMM d'Esterre CD Treurniet KM Niessen WJ Najm M Goyal M. Added value of multiphase CTA imaging for thrombus perviousness assessment. Neuroradiology. (2018) 60:71–9. 10.1007/s00234-017-1907-y 28963573 PMC5748434

[31] Heo JH Nam HS Kim YD Choi JK Kim BM Kim DJ. Pathophysiologic and therapeutic perspectives based on thrombus histology in stroke. J Stroke. (2020) 22:64–75. 10.5853/jos.2019.03440 32027792 PMC7005358

[32] Guglielmi V LeCouffe NE Zinkstok SM Compagne KCJ Eker R Treurniet KM. Collateral circulation and outcome in atherosclerotic versus cardioembolic cerebral large vessel occlusion. Stroke. (2019) 50:3360–8. 10.1161/STROKEAHA.119.026299 31658903 PMC7597992

