# Supplementary material for: Influence of Onset to Imaging Time on Radiological Thrombus Characteristics in Acute Ischemic Stroke
Source: Front Neurol. 2021 Jun 18;12:693427. doi: 10.3389/fneur.2021.693427 (PMC8253046; doi:10.3389/fneur.2021.693427)
Supplement: Supplementary file 1 [file Data_Sheet_1.PDF]

## SUPPLEMENTARY MATERIAL

| **Observed stroke onset (n=187)** | | | | | | |
|---|---|---|---|---|---|---|
| **Outcome variable** | Model 0<br>*Unadjusted* | | Model 1<br>*Adjusted for pre-specified variables** | | Model 2<br>*Adjusted for pre-specified variables* + variables of interest#* | |
| | *β* | 95% CI | *β* | 95% CI | *β* | 95% CI |
| **Thrombus length** | 0.004 | -0.003-0.011 | 0.005 | -0.002-0.011 | 0.005 | -0.002-0.012 |
| **Perviousness** | 0.005 | -0.012-0.021 | 0.006 | -0.011-0.023 | -0.004 | -0.018-0.019 |
| **Thrombus density** | 0.044 | -0.064-0.152 | 0.044 | -0.065-0.153 | 0.084 | -0.031-0.199 |
| **Last seen well (n=58)** | | | | | | |
| **Outcome variable** | Model 0<br>*Unadjusted* | | Model 1<br>*Adjusted for pre-specified variables** | | Model 2<br>*Adjusted for pre-specified variables* + variables of interest#* | |
| | *β* | 95% CI | *β* | 95% CI | *β* | 95% CI |
| **Thrombus length** | -0.001 | -0.008-0.005 | -0.001 | -0.008-0.006 | -0.001 | -0.009-0.007 |
| **Perviousness** | -0.008 | -0.031-0.016 | -0.011 | -0.034-0.013 | -0.015 | -0.039-0.008 |
| **Thrombus density** | 0.142 | -0.034-0.317 | 0.044 | -0.124-0.212 | 0.040 | -0.149-0.228 |

**Supplementary Table 1:** beta coefficients of the effect of time from stroke onset to imaging on thrombus characteristics. Subgroup analysis for patients with observed onset of symptoms (upper) versus a time of last seen well (lower). *CI, confidence interval.* *age, sex, history of atrial fibrillation, intravenous alteplase administration, antiplatelet use. #observed stroke onset, collateral score, direct presentation at thrombectomy center or transfer, stroke etiology (cardio-embolic vs large artery atherosclerosis vs unknown).

| **Patients that received intravenous alteplase (n=188)** | | | | | | |
|---|---|---|---|---|---|---|
| **Outcome variable** | Model 0<br>*Unadjusted* | | Model 1<br>*Adjusted for pre-specified variables** | | Model 2<br>*Adjusted for pre-specified variables* + variables of interest#* | |
| | *β* | 95% CI | *β* | 95% CI | *β* | 95% CI |
| **Thrombus length** | 0.003 | -0.003-0.009 | 0.003 | -0.002-0.009 | 0.003 | -0.004-0.010 |
| **Perviousness** | 0.004 | -0.011-0.018 | 0.005 | -0.010-0.020 | 0.003 | -0.013-0.019 |
| **Thrombus density** | 0.006 | -0.093-0.105 | 0.016 | -0.082-0.114 | 0.058 | -0.054-0.170 |
| **Patients that did not receive intravenous alteplase (n=57)** | | | | | | |
| **Outcome variable** | Model 0<br>*Unadjusted* | | Model 1<br>*Adjusted for pre-specified variables** | | Model 2<br>*Adjusted for pre-specified variables* + variables of interest#* | |
| | *β* | 95% CI | *β* | 95% CI | *β* | 95% CI |
| **Thrombus length** | 0.002 | -0.007-0.010 | 0.002 | -0.005-0.010 | 0.002 | -0.007-0.012 |
| **Perviousness** | -0.010 | -0.029-0.010 | -0.011 | -0.031-0.010 | -0.020 | -0.049-0.009 |
| **Thrombus density** | 0.122 | -0.033-0.277 | 0.135 | -0.022-0.292 | 0.129 | -0.068-0.326 |

**Supplementary Table 2:** beta coefficients of the effect of time from stroke onset to imaging on thrombus characteristics. Subgroup analysis for patients who did (upper) and did not (lower) receive intravenous alteplase before endovascular treatment. *CI, confidence interval.* *age, sex, history of atrial fibrillation, intravenous alteplase administration, antiplatelet use. #observed stroke onset, collateral score, direct presentation at thrombectomy center or transfer, stroke etiology (cardio-embolic vs large artery atherosclerosis vs unknown).

| Absent-poor collaterals (n=88) | | | | | | |
|---|---|---|---|---|---|---|
| **Outcome variable** | Model 0<br>*Unadjusted* | | Model 1<br>*Adjusted for pre-specified variables** | | Model 2<br>*Adjusted for pre-specified variables* + variables of interest*# | |
| | *β* | 95% CI | *β* | 95% CI | *β* | 95% CI |
| **Thrombus length** | 0.002 | -0.006-0.009 | 0.000 | -0.008-0.008 | -0.002 | -0.011-0.008 |
| **Perviousness** | 0.006 | -0.017-0.030 | 0.004 | -0.020-0.028 | 0.012 | -0.015-0.044 |
| **Thrombus density** | 0.047 | -0.101-0.195 | -0.013 | -0.149-0.123 | 0.047 | -0.120-0.215 |
| **Moderate-good collaterals (n=152)** | | | | | | |
| **Outcome variable** | Model 0<br>*Unadjusted* | | Model 1<br>*Adjusted for pre-specified variables** | | Model 2<br>*Adjusted for pre-specified variables* + variables of interest*# | |
| | *β* | 95% CI | *β* | 95% CI | *β* | 95% CI |
| **Thrombus length** | 0.003 | -0.003-0.009 | 0.003 | -0.003-0.009 | 0.002 | -0.005-0.009 |
| **Perviousness** | -0.005 | -0.022-0.012 | -0.004 | -0.020-0.011 | -0.009 | -0.029-0.010 |
| **Thrombus density** | 0.045 | -0.053-0.143 | 0.043 | -0.058-0.144 | 0.092 | -0.023-0.207 |

**Supplementary Table 3:** beta coefficients of the effect of time from stroke onset to imaging on thrombus characteristics. Subgroup analysis by CT-angiography scored collateral grade 0-1 (absent-poor, upper) versus 2-3 (moderate-good, lower). *CI, confidence interval.* *age, sex, history of atrial fibrillation, intravenous alteplase administration, antiplatelet use. #observed stroke onset, collateral score, direct presentation at thrombectomy center or transfer, stroke etiology (cardio-embolic vs large artery atherosclerosis vs unknown).

| Outcome variable | Model 0<br>*Unadjusted* | | Model 1<br>*Adjusted for pre-specified variables** | | Model 2<br>*Adjusted for pre-specified variables* + variables of interest#* | |
|---|---|---|---|---|---|---|
| **Direct presentation patients (n=155)** | | | | | | |
| | $\beta$ | 95% CI | $\beta$ | 95% CI | $\beta$ | 95% CI |
| **Thrombus length** | 0.003 | -0.004-0.009 | 0.002 | -0.005-0.009 | 0.001 | -0.007-0.009 |
| **Perviousness** | -0.003 | -0.020-0.014 | 0.000 | -0.018-0.017 | -0.001 | -0.021-0.019 |
| **Thrombus density** | 0.017 | -0.093-0.126 | -0.005 | -0.114-0.105 | 0.027 | -0.101-0.156 |
| **Transfer patients (n=90)** | | | | | | |
| Outcome variable | Model 0<br>*Unadjusted* | | Model 1<br>*Adjusted for pre-specified variables** | | Model 2<br>*Adjusted for pre-specified variables* + variables of interest#* | |
| | $\beta$ | 95% CI | $\beta$ | 95% CI | $\beta$ | 95% CI |
| **Thrombus length** | 0.002 | -0.005-0.009 | 0.003 | -0.004-0.010 | 0.002 | -0.006-0.010 |
| **Perviousness** | 0.004 | -0.014-0.022 | 0.006 | -0.013-0.024 | 0.009 | -0.009-0.028 |
| **Thrombus density** | 0.128 | -0.014-0.270 | 0.164 | -0.016-0.311 | **0.184** | **0.022-0.346** |
| **Transfer patients treated with IVT, images from primary stroke center used (n=36)** | | | | | | |
| Outcome variable | Model 0<br>*Unadjusted* | | Model 1$<br>*Adjusted for pre-specified variables** | | Model 2$<br>*Adjusted for pre-specified variables* + variables of interest#* | |
| | $\beta$ | 95% CI | $\beta$ | 95% CI | $\beta$ | 95% CI |
| **Thrombus length** | 0.004 | -0.004-0.012 | - | - | - | - |
| **Perviousness** | 0.008 | -0.010-0.027 | - | - | - | - |
| **Thrombus density** | 0.103 | -0.045-0.250 | - | - | - | - |
| **Transfer patients treated with IVT, images from comprehensive stroke center used (n=41)** | | | | | | |
| Outcome variable | Model 0<br>*Unadjusted* | | Model 1$<br>*Adjusted for pre-specified variables** | | Model 2$<br>*Adjusted for pre-specified variables* + variables of interest#* | |
| | $\beta$ | 95% CI | $\beta$ | 95% CI | $\beta$ | 95% CI |
| **Thrombus length** | -0.006 | -0.018-0.007 | - | - | - | - |
| **Perviousness** | 0.013 | -0.019-0.044 | - | - | - | - |
| **Thrombus density** | -0.030 | -0.321-0.261 | - | - | - | - |

**Supplementary Table 4:** beta coefficients of the effect of time from stroke onset to imaging on thrombus characteristics. Subgroup analysis for patients presented directly to a thrombectomy-capable center (upper) and patients transferred from a primary center (lower). *CI, confidence interval**age, sex, history of atrial fibrillation, intravenous alteplase administration, antiplatelet use. #observed stroke onset, collateral score, direct presentation at thrombectomy center or transfer, stroke etiology (cardio-embolic vs large artery atherosclerosis vs unknown). $ Adjusted model 2 not applied due to low number of patients in subgroup.

| Cardioembolic stroke (n=79) | | | | | | |
|---|---|---|---|---|---|---|
| **Outcome variable** | Model 0 *Unadjusted* | | Model 1 *Adjusted for pre-specified variables** | | Model 2 *Adjusted for pre-specified variables* + variables of interest#* | |
| | *β* | 95% CI | *β* | 95% CI | *β* | 95% CI |
| **Thrombus length** | 0.002 | -0.005-0.010 | 0.000 | -0.007-0.008 | 0.003 | -0.007-0.013 |
| **Perviousness** | 0.002 | -0.022-0.027 | 0.007 | -0.016-0.031 | 0.002 | -0.031-0.035 |
| **Thrombus density** | 0.077 | -0.056-0.210 | 0.044 | -0.093-0.181 | 0.187 | -0.007-0.381 |
| **Large artery atherosclerosis stroke (n=18)** | | | | | | |
| **Outcome variable** | Model 0 *Unadjusted* | | Model 1$ *Adjusted for pre-specified variables** | | Model 2$ *Adjusted for pre-specified variables* + variables of interest#* | |
| | *β* | 95% CI | *β* | 95% CI | *β* | 95% CI |
| **Thrombus length** | -0.004 | -0.023-0.015 | - | - | - | - |
| **Perviousness** | -0.013 | -0.057-0.030 | - | - | - | - |
| **Thrombus density** | -0.009 | -0.313-0.294 | - | - | - | - |
| **Stroke with unknown cause (n=142)** | | | | | | |
| **Outcome variable** | Model 0 *Unadjusted* | | Model 1 *Adjusted for pre-specified variables** | | Model 2 *Adjusted for pre-specified variables* + variables of interest#* | |
| | *β* | 95% CI | *β* | 95% CI | *β* | 95% CI |
| **Thrombus length** | 0.004 | -0.003-0.010 | 0.004 | -0.002-0.011 | 0.001 | -0.006-0.009 |
| **Perviousness** | 0.003 | -0.012-0.019 | 0.004 | -0.011-0.019 | 0.002 | -0.015-0.019 |
| **Thrombus density** | 0.029 | -0.083-0.140 | 0.041 | -0.072-0.153 | 0.064 | -0.065-0.192 |

**Supplementary Table 5:** beta coefficients of the effect of time from stroke onset to imaging on thrombus characteristics. Subgroup analysis for patients with suspected cardioembolic stroke (upper), stroke likely due to large artery atherosclerosis (middle), and patients with unknown cause of stroke (lower). Three patients had strokes with carotid webs as most likely cause as no other possible cause could be identified. *CI, confidence interval.* *age, sex, history of atrial fibrillation, intravenous alteplase administration, antiplatelet use. #observed stroke onset, collateral score, direct presentation at thrombectomy center or transfer, stroke etiology (cardio-embolic vs large artery atherosclerosis vs unknown). $ Adjusted models not applied due to low number of patients in subgroup.

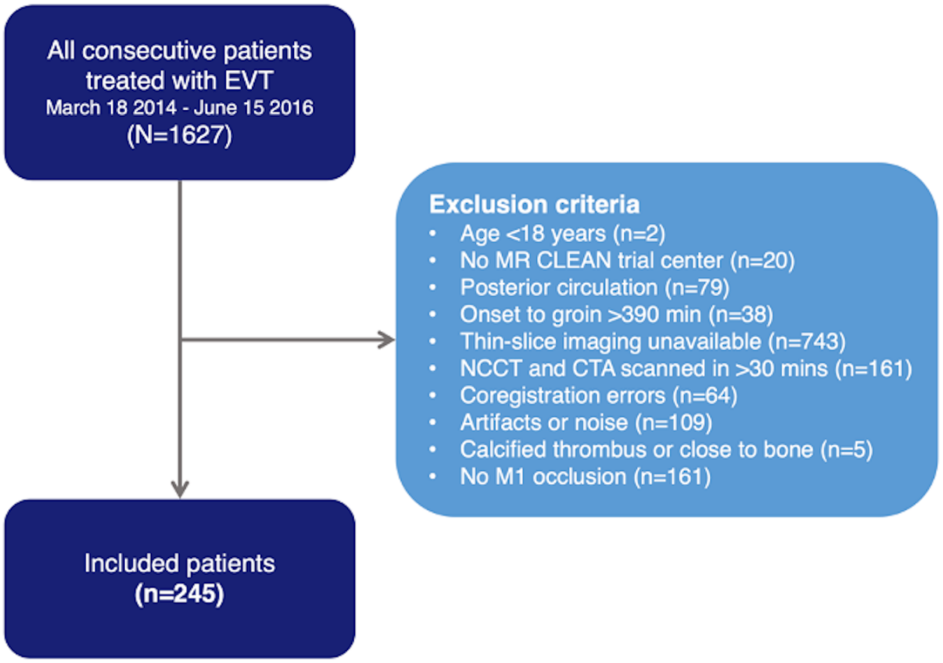


**Supplementary Figure 1**: patient inclusion flowchart. *CTA, CT angiography; EVT, endovascular treatment; mins, minutes; MR CLEAN, Multicenter Randomized Clinical trial of Endovascular Treatment for Acute ischemic stroke in the Netherlands; NCCT, non-contrast CT.*

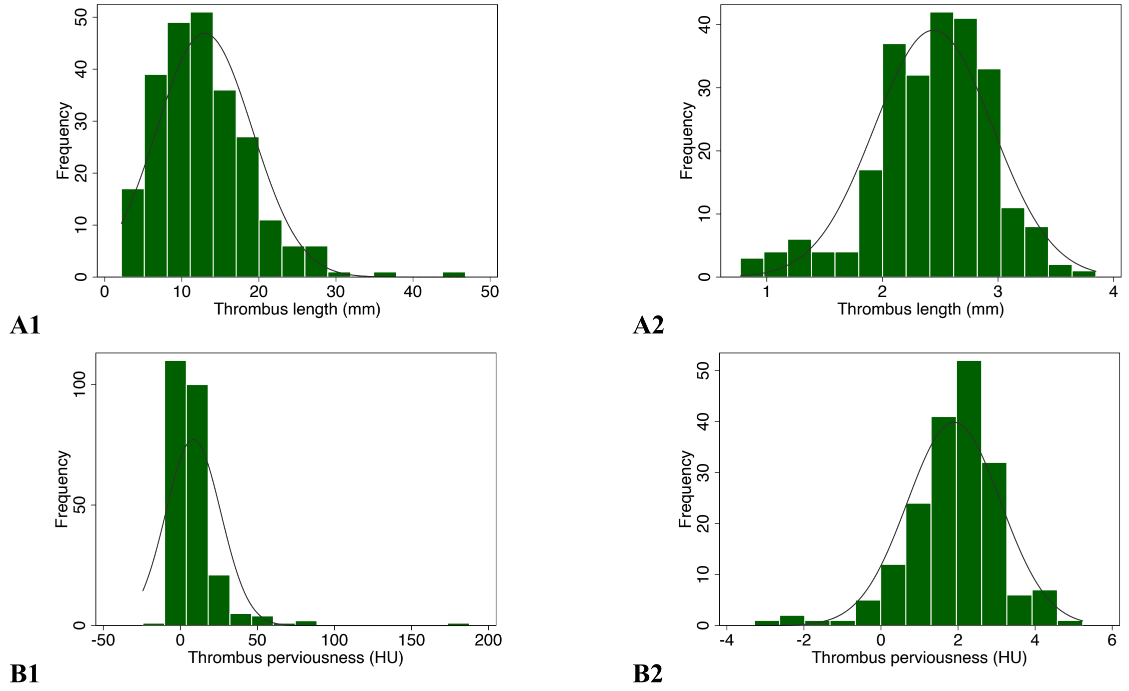


**Supplementary Figure 2**: distribution of outcome data for thrombus length (A) and thrombus perviousness (B) before (1) and after (2) log-transformation. *HU, Hounsfield Units; mm, millimeter.*

**Observed stroke onset (n=187)**

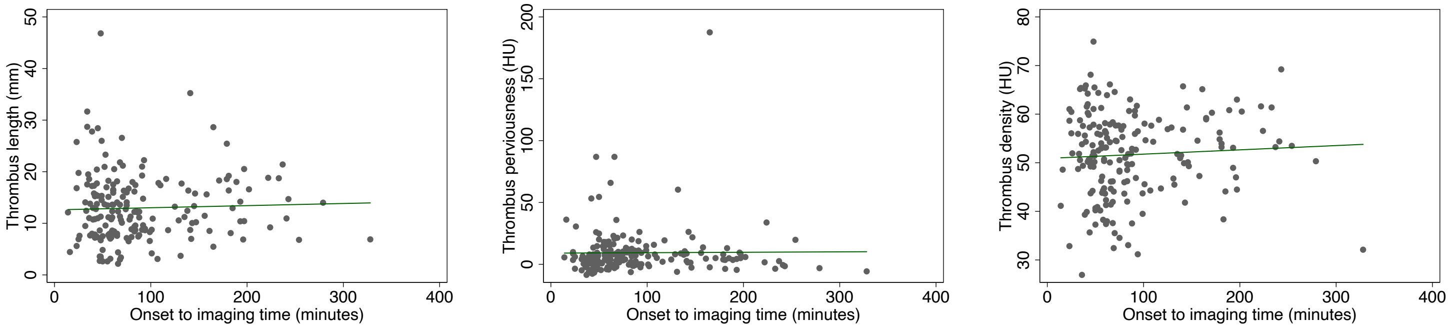


**A1** **B1** **C1**

**Last seen well (n=58)**

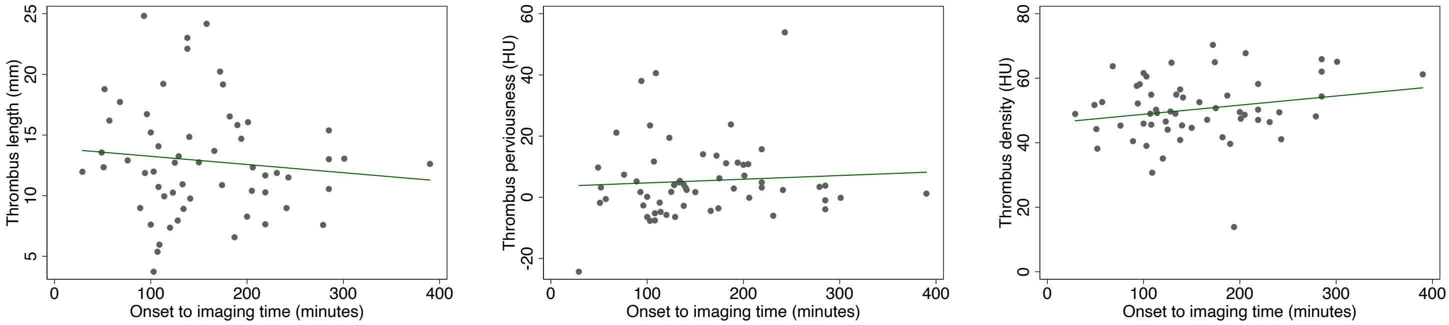


**A2** **B2** **C2**

**Supplementary Figure 3**: scatter plots of imaging-to-onset time and thrombus characteristics, for with observed onset of symptoms (upper row 1) versus time of last seen well (lower row 2). A: time versus thrombus length, B: time versus perviousness, C: time versus non-contrast CT thrombus density. *HU, Hounsfield Units; mm, millimeter.*

**Patients that received intravenous alteplase (n=188)**

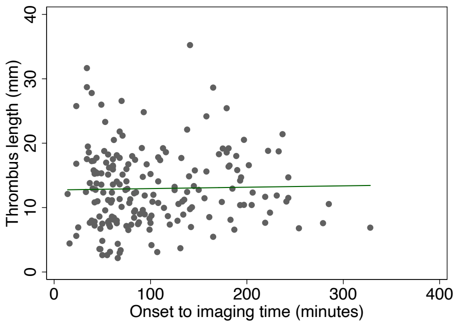


A1

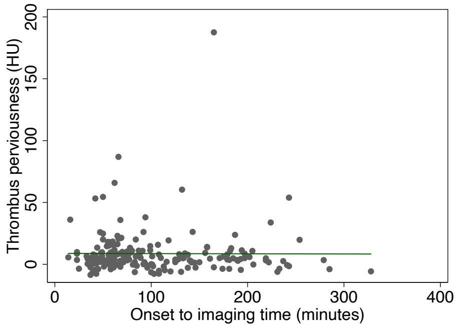


B1

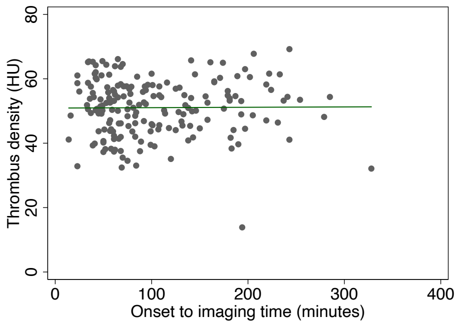


C1

**Patients that did not receive intravenous alteplase (n=57)**

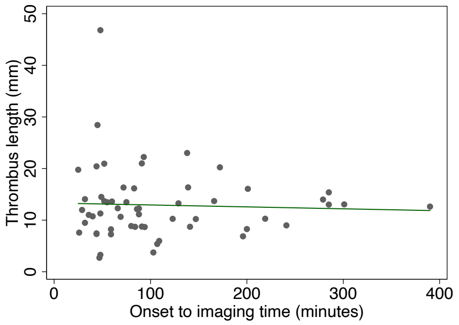


A2

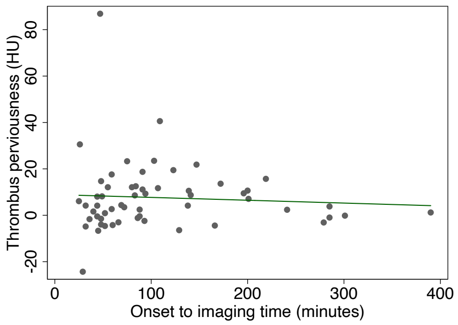


B2

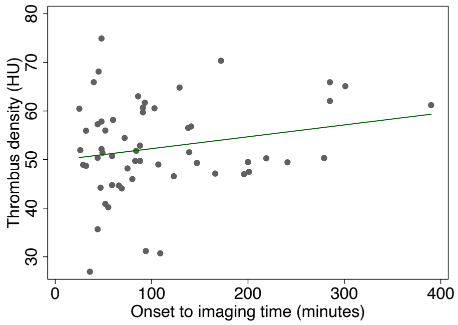


C2

**Supplementary Figure 4**: scatter plots of imaging to onset time and thrombus characteristics, for patients who did (upper row 1) and did not (lower row 2) receive intravenous alteplase before endovascular treatment. A: time versus thrombus length, B: time versus perviousness, C: time versus non-contrast CT thrombus density. *HU, Hounsfield Units; mm, millimeter.*

**Absent-poor collaterals (n=88)**

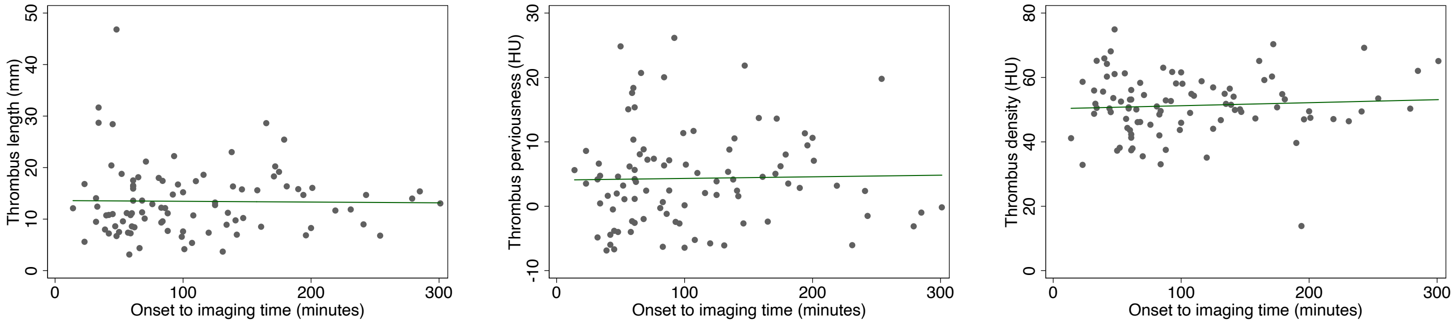


**A1** **B1** **C1**

**Moderate-good collaterals (n=152)**

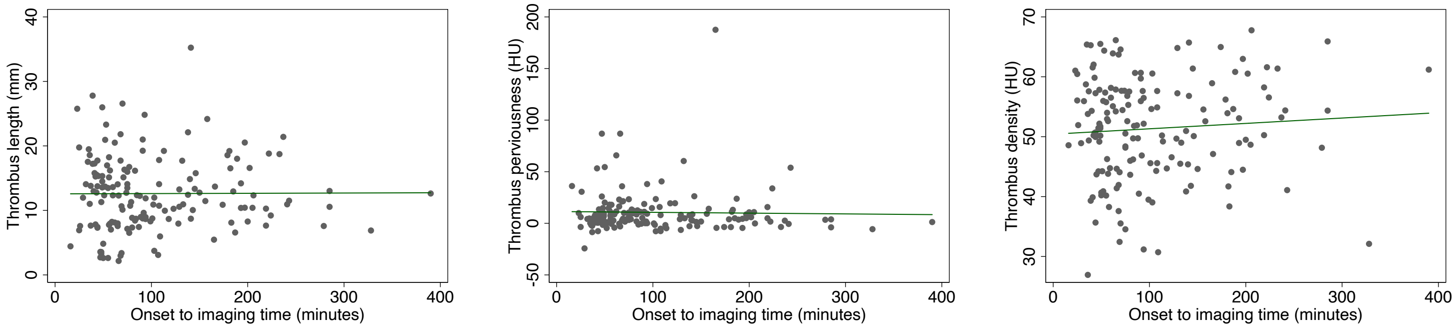


**A2** **B2** **C2**

**Supplementary Figure 5**: scatter plots of imaging to onset time and thrombus characteristics, by CT-angiography scored collateral grade 0-1 (absent-poor, upper row 1) versus 2-3 (moderate-good, lower row 2). A: time versus thrombus length, B: time versus perviousness, C: time versus non-contrast CT thrombus density. *HU, Hounsfield Units; mm, millimeter.*

**Direct presentation patients (n=155)**

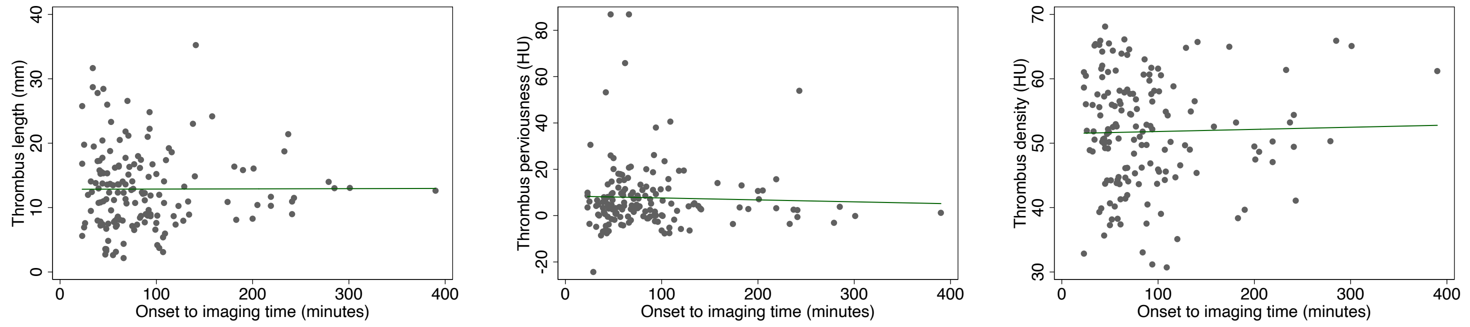


**A1** **B1** **C1**

**Transfer patients (n=90)**

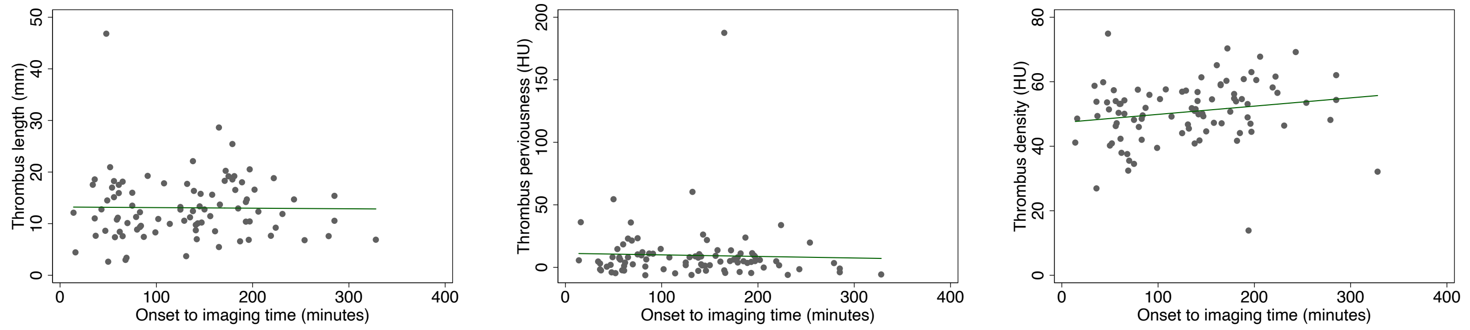


**A2** **B2** **C2**

**Transfer patients treated with IVT, images from primary stroke center used (n=36)**

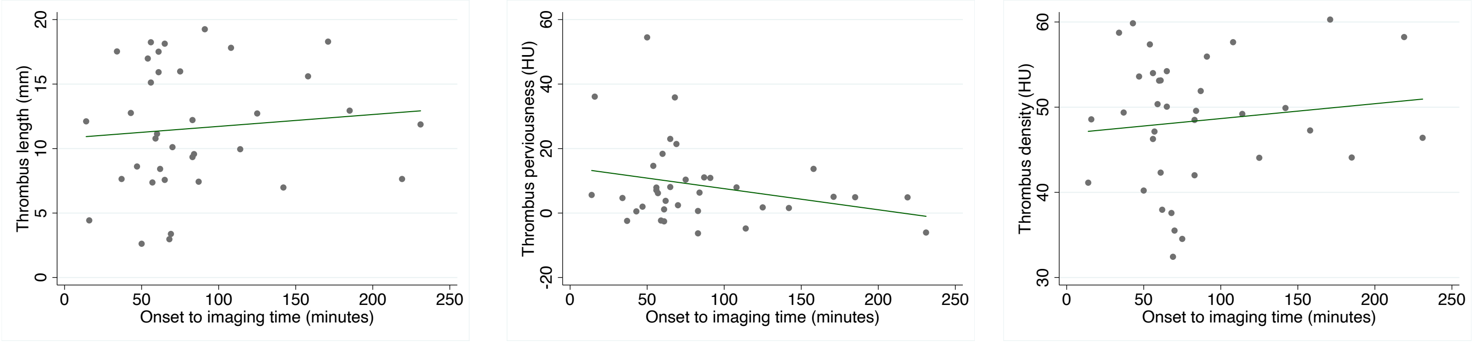


**A3** **B3** **C3**

**Transfer patients treated with IVT, images from comprehensive stroke center used (n=41)**

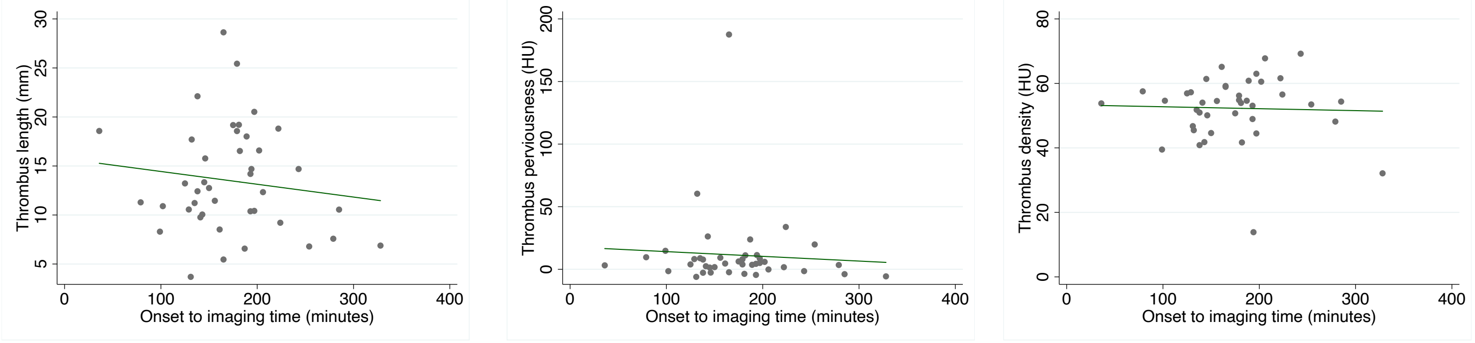


**A4** **B4** **C4**

**Supplementary Figure 6**: scatter plots of imaging to onset time and thrombus characteristics, for patients presented directly to a thrombectomy-capable comprehensive stroke center (upper row 1) and patients transferred from a primary stroke center (lower row 2). In

addition, a subgroup analysis of transfer patients treated with IVT for whom the primary center images were used (row 3), compared to those for whom comprehensive center images were used, leaving more time for IVT to break down thrombi (row 4). A: time versus thrombus length, B: time versus perviousness, C: time versus perviousness, D: time versus non-contrast CT thrombus density. *HU, Hounsfield Units; IVT, intravenous alteplase; mm, millimeter.*

**Cardioembolic stroke (n=79)**

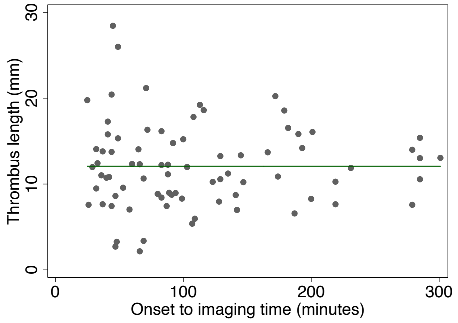


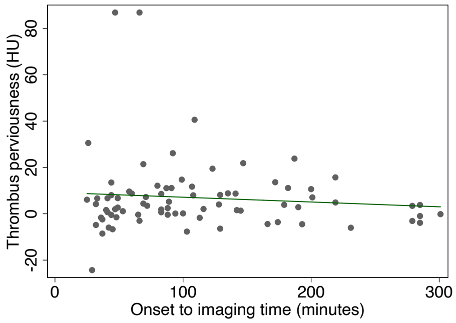


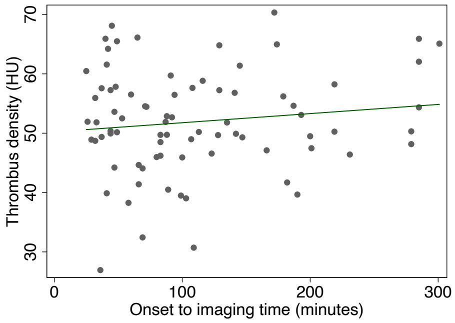


**A1** **B1** **C1**

**Large artery atherosclerosis stroke (n=18)**

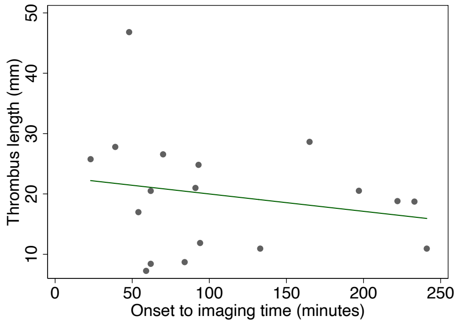


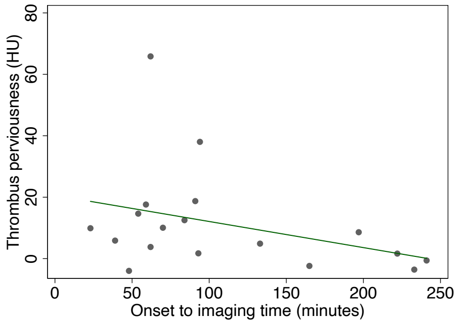


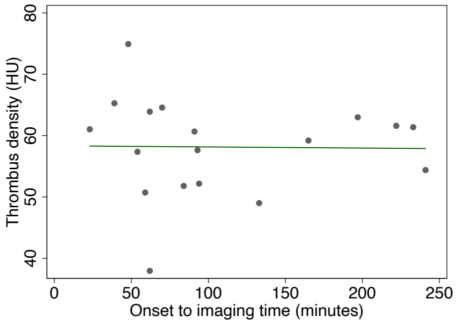


**A2** **B2** **C2**

**Stroke with unknown cause (n=142)**

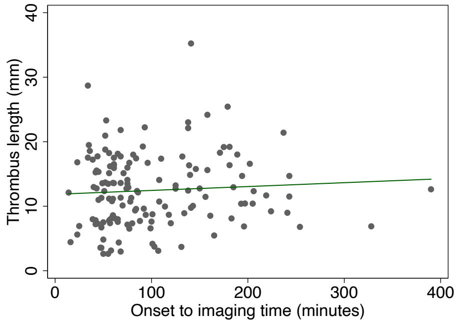


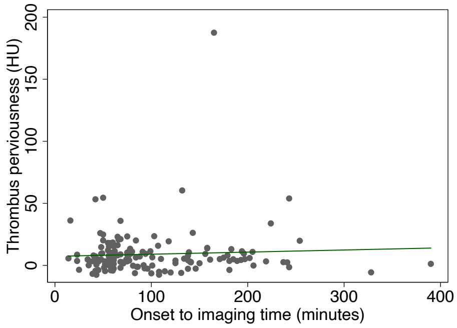


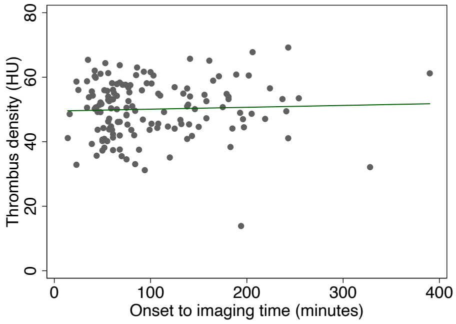


**A3** **B3** **C3**

**Supplementary Figure 7**: scatter plots of imaging to onset time and thrombus characteristics, for patients with suspected cardioembolic stroke (upper row 1), stroke suspected due to large artery atherosclerosis (middle row 2), and stroke with unknown cause (lower row 3). A: time versus thrombus length, B: time versus perviousness, C: time versus perviousness, D: time versus non-contrast CT thrombus density. *HU, Hounsfield Units; mm, millimeter.*
